# P-283. Epidemiology, clinical, phenotypic, and genotypic characteristics of multidrug, extensive and pan-drug resistant *Acinetobacter baumannii* from Qatar

**DOI:** 10.1093/ofid/ofae631.486

**Published:** 2025-01-29

**Authors:** Hamad Abdel Hadi

**Affiliations:** Communicable Diseases Centre, Hamad Medical Corporation , Doha, Ad Dawhah, Qatar

## Abstract

**Background:**

Multidrug-resistant *Acinetobacter baumannii-calcoaceticus* complex is associated with significant morbidity and mortality with limited treatment options. The outlined study aimed to evaluate the epidemiology , clinical , phenotypic and genotypic characteristics of multidrug resistant (MDR) Acinetobacter species from Qatar between 2017-2020 .

**Figure:**
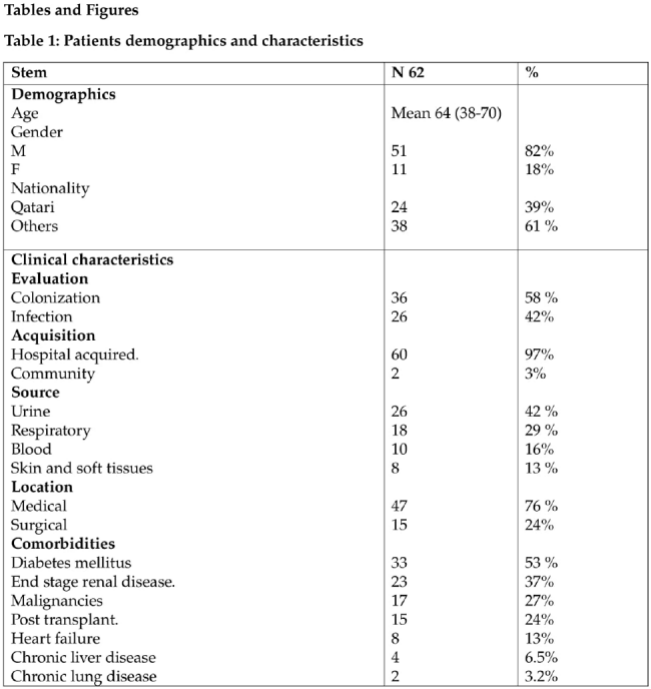

**Methods:**

A prospective study was conducted between 2017-2020 for all clinical isolates of Acinetobacter species. Microbial identifications and antimicrobial susceptibility tests were performed using automated MALDI-TOF MS and BD Phoenix^TM^ system according to the manufacturer’s recommendations while additional ASTs were performed with E test . Genomic tests were performed using whole genome sequence

**Figure:**
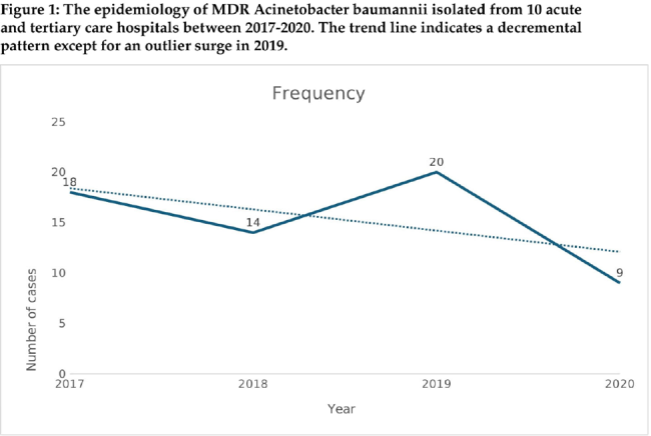

**Results:**

Out of 536 isolates, 62 (11.5%) resistant isolates were identified showing decremental trend over the study period with dominance of sequence types: ST2 (71 %), ST 25 % (8%) and ST 164 (8%). Main age of acquisition was 64 year (38-70). Isolate sites were urinary (42 %), respiratory (29%), bloodstream (16%), skin and soft tissues (13%) mainly acquired at hospitals (97 %). Existing multiple comorbidities and previous antimicrobial exposure were present in most cases. Multiple antimicrobial regimes for management were dominated by colistin, meropenem, amikacin, cotrimoxazole and tigecycline with 30-day mortality of 16 %. Microbiological evaluation demonstrated extensive resistance ranging between 85-100 % for active agents including meropenem and ampicillin-sulbactam at 97.4 % as well as for novel agents: ceftazidime-avibactam (100%), ceftolozane-tazobactam, meropenem-vaborbactam and imipenem- relebactam at 97.4 %. All ST2 isolates carried class D *bla*_OXA-23_ and *bla*_OXA-51_ family, that confer resistance to carbapenems, as well 7 isolates carried class B MBL *bla*_NDM-1_.

**Figure:**
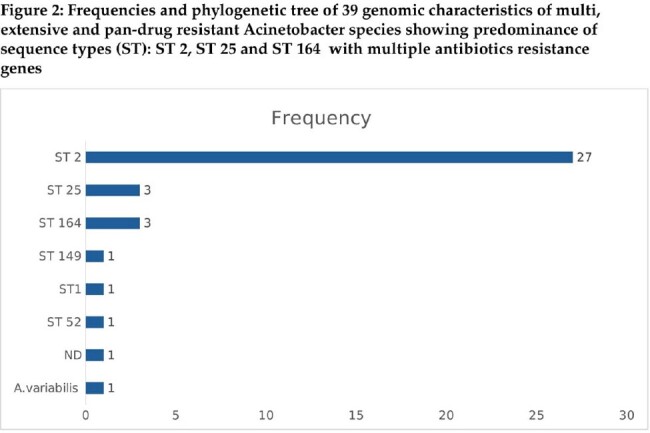

**Conclusion:**

Acquisition of resistant *Acinetobacter baumannii* is infrequent with decremental trend dominated by ST 2, mainly isolated from the elderly with multiple comorbidities and risk factors. Predominant isolation sites are urinary, respiratory, bloodstream, skin and soft tissues. Extensive resistance profiles limit treatment options for conventional and novel agents pointing towards urgent needs for alternative therapy.

**Figure:**
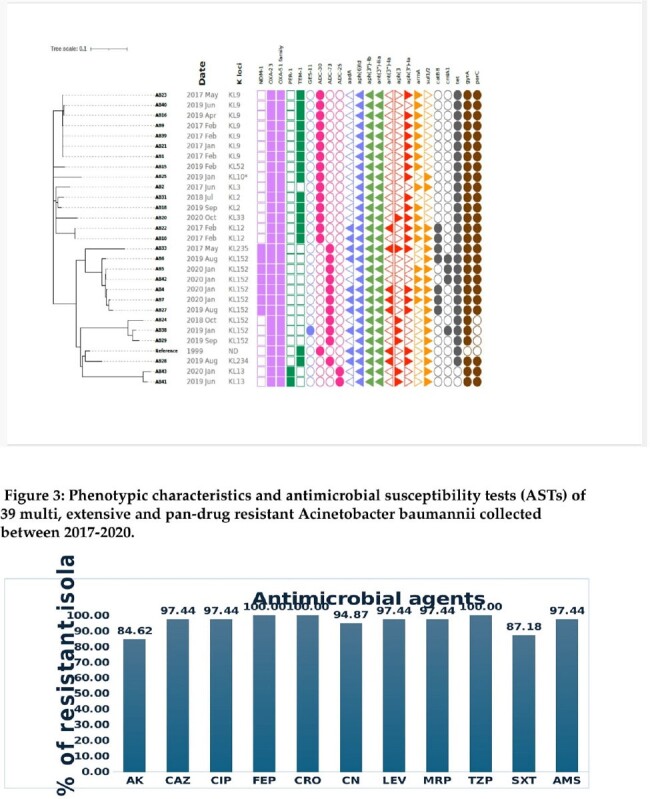

**Disclosures:**

**All Authors**: No reported disclosures

